# Effect of Furostanol Saponins from Allium Macrostemon Bunge Bulbs on Platelet Aggregation Rate and PI3K/Akt Pathway in the Rat Model of Coronary Heart Disease

**DOI:** 10.1155/2019/9107847

**Published:** 2019-06-23

**Authors:** Hui Feng, Zhipeng Wang, Changsong Wang, Xinyi Zhu, Zhigang Liu, Hongmei Liu, Ming Guo, Qian Hou, Zhenrong Chu

**Affiliations:** Department of Traditional Chinese Medicine, Zhongda Hospital Affiliated to Southeast University, Nanjing, China

## Abstract

*Aim*. To investigate the effect of Furostanol Saponins from Allium Macrostemon Bunge Bulbs (FSAMB) on platelet aggregation rate of rats with coronary heart disease and discuss the mechanism of FSAMB affecting the platelet aggregation rate through PI3K/Akt pathway. We established the rat models with coronary heart disease (CHD) and prepared the platelet-rich plasma. The effect of different concentrations of FSAMB on platelet aggregation in SD rats induced by ADP was observed in vitro and in vivo. And Lactate Dehydrogenase (LDH), Creatine Kinase-MB Form (CK-MB), and Cardiac Troponin I (cTnI) are detected in the blood to know the level of damage to heart cells. The expansion of platelets in the immobilized fibrinogen in different concentrations of FSAMB was observed. Western blot was conducted to detect the phosphorylation level of protein kinase B (also known as Akt) and the expression level of phosphoinositide 3-kinase (PI3K). We found that FSAMB had a significant inhibitory effect on the ADP-induced platelet aggregation in vitro. Intragastric administration of FSAMB also inhibited platelet aggregation induced by ADP in rats. LDH, CK-MB, and cTnI levels in serum of rats in FSAMB (672 mg/kg) group were lower than those in the model control group after the intervention (P<0.01 or P<0.05). FSAMB inhibited the expansion of platelets on immobilized fibrinogen. Also, FSAMB inhibited ADP-induced platelet PI3K expression and Akt phosphorylation. The inhibition of Akt phosphorylation by FSAMB was more obvious after the inhibition of the expression of PI3K. This study demonstrated that FSAMB can reduce the degree of myocardial cell damage and inhibit ADP-induced platelet aggregation in SD rats, possibly by inhibiting platelet PI3K/Akt signaling pathway in vitro and in vivo.

## 1. Introduction

Platelets are granules that are circulated in the blood without a nucleus. Their primary function is to participate in stopping bleeding and forming thromboses. There are a variety of receptors on the platelet membrane which serve as the primary tools for transmitting external signals into platelets. They determine the reactivity of platelets to different agonists and adhesion proteins. Surface receptors such as tyrosine kinase receptor, integrin family, seven-layer transmembrane receptor, C-type lectin receptor family, and Leucine-Rich Repeat family and internal particles such as adenosine diphosphate(ADP), thromboxane A2 (thromboxane A2, TXA2), and 5-hydroxytryptamine (5-HT) largely determine the cellular characteristics of platelets. Unlike other nucleated cells, platelets lack a nucleus and therefore cannot rely on gene regulation and protein synthesis to respond to changes in the external environment. Although there exist some evidence showing that the residual mRNA from macrophages can synthesize proteins, a large number of recent studies have shown that platelets rely mainly on multiple receptor signaling to cope with different physiological conditions and pathological changes [1, 2]. Platelets play an essential role in the processes of coronary atherosclerotic heart disease and cerebral infarction [3]. These receptors and signaling pathways are of great significance in platelet adhesion and release.

Allium Macrostemon Bunge (Chinese phonetic: XièBái, AMB) is an essential medicine for treating chest pain and heart pain. It has the effect of activating qi circulation and promoting digestion, activating yang, and removing stasis in Traditional Chinese Medicine (TCM) theory. Treatise on Febrile Diseases and the Chinese Pharmacopeia (2011 version) [4] mentioned prescriptions like Jiawei Gualou Xiebai Baijiu Decoction, Gualou Xiebai Banxia Decoction, Zhishi Xiebai Guizhi Decoction, which are famous and often used in clinical practice in treating chest pain and heart pain. Studies have shown that FSAMB compounds participate in the whole process of coronary atherosclerosis in ways of antiplatelet aggregation, antioxidation, and lowering blood fat [5]. However, its microscopic mechanism has not been explained. What kind of signaling pathway affects the adhesion and release of platelets by FSAMB compounds remains to be studied. Therefore, our group explored the intervention of FSAMB on platelet activating factor-induced platelet aggregation and its effect on surface glycoprotein levels.

## 2. Materials and Methods

### 2.1. Animals

Specific pathogen-free (SPF) 7-8-week-old Sprague-Dawley (SD) rats (with body weight ranging from 221 to 310g) were used in the present study, which were purchased from Western Biotech, Chongqing, China. All rats were provided with free access to food and water in an air-conditioned room (25°C and 65% humidity). All the rats were acclimatized for one week to reduce the stress response caused by environmental changes. This study was performed in accordance with the guidance of Care and Use of Laboratory Animals of HNI and was approved by the Independent Ethics Committee for Clinical Research of Zhongda Hospital affiliated to Southeast University.

### 2.2. Model Establishment

After acclimatization for one week, the rats needed to be modeled were fed with high-fat diet containing 2% cholesterol, 0.5% sodium cholate, 3% lard oil, 0.2% propylthiouracil, and 94.3% basic diet with vitamin D3 (1.25 × 106 *μ*/kg) for three months [6]. On three days before the last feeding, these rats were injected with postcolumn voxel (30 U/kg) once a day for three consecutive days.

### 2.3. Drugs and Equipment

FSAMB, Allii Macrostemonis Bulbus, is a bulb of Allium Macrostemon Bunge or A. Chinese G. Don, which is mainly produced in northeast China and northern China. It is also planted in Zhejiang, Hubei, and other provinces. This Traditional Chinese Medicine has dual chemical compounds such as sulfur, nitrogen, and saponins [7, 8]. The FSAMB in this experiment was a mixture of Furostanol Saponins, which were extracted from Allium Macrostemon Bunge Bulbs. The extraction method is as follows: fresh Allium Macrostemon Bunge (Nanjing Crane Age Pharmaceutical Service Co., Ltd., origin: Pingdingshan City, Henan Province) is mashed, and the total saponin is extracted from the ethanol first, and then six mixtures of Furostanol Saponins from Allium Macrostemon Bunge Bulbs with known chemical structure were separated by chromatography [9]. Clopidogrel Hydrogen Sulphate Tablets (Batch number J20130083, 75mg/tablet) were purchased from Sanofi Winthrop Industrie France. Aspirin Enteric-coated Tablets (Batch number J20130078, 100mg/tablet) were purchased from Bayer Health Care Manufacturing S.r.l.

ADP, phalloidin, PGE1, fibrinogen, apyrase, and LY294002 were bought from Sigma-Aldrich. Phosphatidylinositol 3-kinase (phosphoinositide 3-kinase, PI3K) p110*β* I anti-phosphorylation, Akt (Ser 473) I antibody, Akt I antibody, GAPDH I antibody, and horseradish peroxidase-labeled goat anti-rabbit IgG II antibody were bought from ABGENT. West Pico chemiluminescent substrate was bought from Pierce Biotechnology. PVDF membrane was purchased from Shanghai Solarbio Bioscience & Technology Co., Ltd. Bioluminescence agglutination tester was purchased from Chrono-Log Corporation, Havertown. Gel Imager Imaging (JS-2000) was purchased from P&Q Science and Technology. The fluorescence microscope was purchased from Quantum Design US. High-speed centrifuge (model: GL10MA) was made by Hunan Kaida Scientific Equipment Co., Ltd. Microplate Oscillator (model MPS40) was made by Shanghai Brave Construction Development Co., Ltd.

### 2.4. Determination of In Vitro Platelet Aggregation Rate

Forty-eight rats were numbered and randomly divided into six groups by their weight and sex, namely, normal control group, model control group, FSAMB low-dose group, FSAMB middle-dose group, FSAMB high-dose group, and drug control group, with 8 rats in each group. The normal control group was fed with the basal diet. Other groups began to build models for 3 months. Three months later, all the rats were anesthetized by intraperitoneal injection of 10% chloral hydrate (0.35 g/kg), and blood was taken by an abdominal aortic puncture. 3.2% sodium citrate solution and blood were mixed with a ratio of 1:9 to achieve anticoagulation. PGE1 (final concentration 0.1 mg/L) was added to the mixed whole blood, and the whole blood was mixed and then centrifuged at 300×g for 6 minutes. The upper layer of plasma was aspirated. PGE1 (final concentration 0.1 mg/L) was added and centrifuged at 900×g for 10 minutes, and the supernatant was discarded to obtain a concentrated platelet mass, and appropriate amounts of tyrode buffer (12 mmol/L NaHCO_3_, 138 mmol/L NaCl, 5.5 mmol/L glucose, 2.9mmol/L KCl, 2mmol/L MgCl_2_, 0.42mmol/L NaH_2_PO_4_, and 10mmol/L HEPES, pH7.4) and PGE1 (final concentration 0.1mg/L) were added. It was blown gently with a Pasteur pipette and resuspend platelets and centrifuged at 900×g for 5 minutes; the supernatant was discarded; platelet mass was obtained; the platelet pellet in tyrode buffer containing 2×10-5 U/L apyrase was resuspended; and the final platelet concentration was adjusted to 4×1011/L. Low, medium, and high doses of FSAMB (168mg/kg, 336mg/kg, and 672mg/kg) and aspirin were added, and the platelet aggregation rate was measured by the Born method as described in the literature [10, 11]. The maximum aggregation rate was monitored with a bioluminescence agglutination meter.

### 2.5. Determination of In Vivo Platelet Aggregation Rate

Forty-eight rats were numbered and randomly divided into six groups by their weight and sex, namely, normal control group, model control group, FSAMB low-dose group, FSAMB middle-dose group, FSAMB high-dose group, and drug control group, with 8 rats in each group. The normal control group was fed with the basal diet. Other groups began to build models for 3 months. After successful modeling, FSAMB low-, medium-, and high-dose groups were given 168mg/kg, 336mg/kg, and 672mg/kg FSAMB, respectively, and drug control group (clopidogrel) was given clopidogrel 30mg/kg by intragastric administration once a day for 7 days. The normal control group and model control group were given an equal volume of normal saline once a day for 7 days. One hour after the last administration, blood was taken from the abdominal aorta, and arterial blood used for antiplatelet aggregation experiments was anticoagulated with 3.8% sodium citrate. Lactate dehydrogenase (LDH), Creatine Kinase-MB Form (CK-MB), and Cardiac Troponin I (cTnI) were detected in the blood to know the level of damage to heart cells. While platelet-rich plasma (PRP) and platelet-poor plasma (PPP) were obtained by centrifugation at 180 × g and 1000 × g for 10 minutes, PRP was centrifuged at 1000 × g for 10 minutes, and then the cells were resuspended in PBS (containing 1.0% bovine serum albumin). After washing with 1 mmol/L CaCl2), the cell viability was observed to be higher than 95% by trypan blue exclusion test, and the cell concentration was adjusted to 4×10^11^/mL. PRP and PPP were prepared before administration and 60, 120, 180, and 240 minutes, respectively, after administration. ADP-induced platelet aggregation was monitored by bioluminescence agglutination meter.

### 2.6. Platelet Expansion on Fibrinogen

200um thick clean coverslips were coated with 50 mg/L fibrinogen (200 uL) at 4°C for 12h, and then the coverslips were washed twice with PBS; then 1% bovine serum albumin solution was added and the coverslips were put under 25°C for 1 h and then the blocking solution was removed. Different concentrations of FSAMB were added to 4×10^11^/L platelets for 5 minutes. The platelets were dropped on fibrinogen-coated coverslips and incubated in a 5% CO_2_ incubator at 37°C for 45 minutes. The coverslips were gently washed 3 times with PBS and then were fixed with 4% paraformaldehyde for 20 minutes and then gently washed 3 times with PBS again. 200 uL of phalloidin (1mg/L) was added in the dark and stored in the dark for 60 minutes, and the phalloidin was removed and washed three times with PBS, and the adhered platelets were observed with a fluorescence microscope.

### 2.7. Western Blot Detection

Immediately after the platelet aggregation reaction, the washed platelets were placed on ice to terminate the reaction and centrifuged at 3000×g for 3 minutes to obtain platelet pellet and washed twice with PBS. Appropriate amount of cell lysate was added and lysed on ice for 30 minutes and centrifuged at 12000×g for 10 minutes, and the supernatant was obtained, which was the total cell protein. 20*μ*L of the lysed sample was taken for BCA protein quantification, and the remaining samples were added to the 5×loading buffer, boiled at 100°C for 5 minutes, and stored at -80°C. 40*μ*g of the sample was taken, and the sample was electrophoresed, transferred, and sealed with 5% BSA for 1 h at room temperature, and the membrane was washed 3 times. Specific antibodies (PI3K p110*β*, Akt, p-Akt^Ser473^, and GAPDH) were added, respectively, and incubated for 1 h at room temperature with gentle shaking and then incubated overnight at 4°C in a refrigerator. After washing the membrane 3 times, the II antibody was added, and the membrane was incubated for 1 hour at room temperature, and the membrane was washed 3 times. The images were got with the Gel Imager.

### 2.8. Statistical Processing

The analysis was performed by SPSS18.0 statistical software. Measurement data was represented by $\overset{-}{X}+S$. The comparison was made by* t*-test. Count data was expressed in percentage (%). One-Way ANOVA was adopted in the analysis and comparison. LSD method was used in multiple comparisons. And inspection standard (*α*) was 0.05.

## 3. Results

### 3.1. Influence of FSAMB on Platelet Aggregation

It can be seen from Table 1 that LDH, CK-MB, and cTnI of normal cardiomyocytes are at a low level (normal control group), and these indexes are at a high level in the rats after modeling (P<0.01), and then the rat model of coronary heart disease is established successfully. Under the in vitro condition, 10*μ*mol/L ADP could induce strong aggregation of washed platelets, and the maximum aggregation rate within 5 minutes was 73.1±4.1%. After washing the platelets with 168mg/kg, 336mg/kg, and 672mg/kg FSAMB, the platelet aggregation rate (PAR) was significantly reduced. The corresponding platelet aggregation rate was statistically significant compared with the model control group (P<0.01). The platelet aggregation rates of 336mg/kg and 672mg/kg FSAMB group were significantly lower than those of the aspirin group, and the difference was statistically significant (P<0.01 or P<0.05).

Under the in vivo conditions, different concentrations of FSAMB (168 mg/kg, 336 mg/kg, and 672 mg/kg) significantly inhibited platelet aggregation induced by ADP, and the difference was statistically significant compared with the model control group (P<0.01), and the inhibition was dose-dependent. The inhibitory effect of FSAMB at the high doses on platelet aggregation induced by ADP was comparable to that of clopidogrel (P>0.05) in Table 1.

Under the in vivo conditions, LDH, CK-MB, and cTnI levels in serum of rats in FSAMB (672 mg/kg) group were lower than those in the model control group after the intervention (P<0.01 or P<0.05), while LDH and CK-MB levels in serum of rats in drug control group (clopidogrel) were lower than those in the model control group (P<0.01) in Table 1.

### 3.2. Effect of FSAMB on the Rat Platelet Spreading in Immobilized Fibrinogen

**Figure 1:**
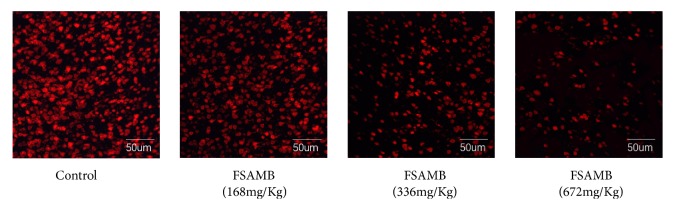
FSAMB inhibited rat platelet spreading on immobilized fibrinogen. The platelet spreading was visualized under a fluorescence microscope. Control: model control group.

As shown in Figure 1 and Table 2, platelets were completely spreading after 45 minutes on immobilized fibrinogen, and the percentage of spreading area was 30.51±4.08%. When treated with 168*μ*mol/L, 336*μ*mol/L, and 672*μ*mol/L FSAMB for 5 minutes, the spreading on fibrinogen was significantly inhibited and dose-related (P<0.01), and its spreading area percentage, compared with the model control group, decreased to 16.09±2.36%, 12.32±2.14%, and 10.13±1.40%, respectively (Figure 1 and Table 2).

### 3.3. Effect of FSAMB on PI3K Expression and Akt Phosphorylation

**Figure 2:**
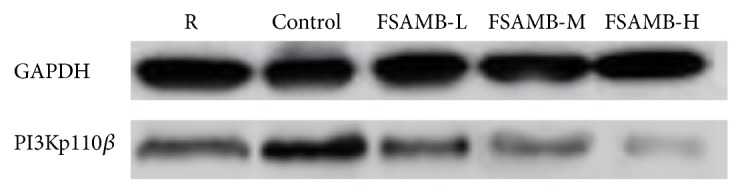
FSAMB inhibited PI3K expression in ADP- (10*μ*mol/L) stimulated platelets. R: resting platelets. Control: model control group.

**Figure 3:**
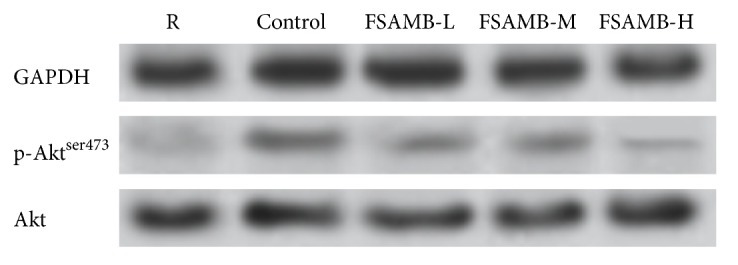
FSAMB inhibited Akt phosphorylation in ADP- (10*μ*mol /L) stimulated platelets. R: resting platelets. Control: model control group.

ADP-stimulated platelet activation could significantly increase PI3K protein levels, and FSAMB could significantly inhibit PI3K expression (P<0.01). When ADP stimulates platelets, it increases Akt phosphorylation, and FSAMB could significantly inhibit Akt phosphorylation (P<0.01) (Figures 2 and 3 and Table 3).

### 3.4. Effect of PI3K Inhibitor LY294002 on Akt Phosphorylation

**Figure 4:**
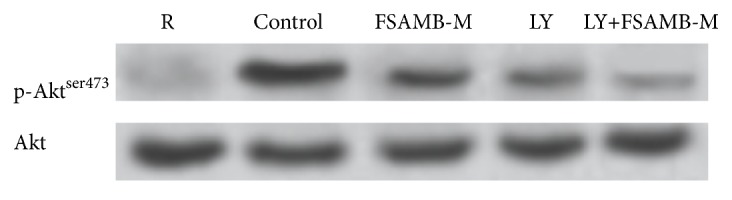
Effect of PI3K inhibitor LY294002 on Akt phosphorylation. R: resting platelets. Control: model control group. LY: LY2294002.

We used the PI3K inhibitor (LY294002) to further confirm the effect of FSAMB on PI3K/Akt activation. The results showed that FSAMB or LY294002 used alone could not inhibit Akt phosphorylation completely, whereas the inhibition of Akt phosphorylation could be strengthened when FSAMB was used together with LY294002, indicating that FSAMB and PI3K inhibitors could inhibit Akt phosphorylation synergistically (Figure 4 and Table 4).

## 4. Discussion

Allium Macrostemon Bunge, which was used as a common Chinese medicine for the treatment of coronary heart disease, has a history of thousands of years in Treatise on Febrile and Miscellaneous Disease. In recent years, studies have shown that FSAMB compounds participate in the whole process of coronary atherosclerosis in ways of antiplatelet aggregation, antioxidation, and lowering blood fat [5]. Our research finds that high concentrations of FSAMB can reduce LDH and CK-MB levels in serum of rat, and this effect is similar to that of clopidogrel. Also, high concentrations of FSAMB can reduce cTnI levels, while the effect of this is not available in clopidogrel. This suggests that FSAMB can reduce the degree of myocardial cell damage in the occurrence of coronary heart disease. The specific mechanism of action is further studied in this research.

ADP is the most important substance in the body to induce platelet aggregation and is present in high-density particles in platelet cells. ADP's ability to activate platelet aggregation is weak; it mainly causes platelet aggregation by binding to the ADP receptor on the platelet membrane. When platelets are activated, ADP is released from dense particles of platelets, which affects the shape and biological behavior of platelets by ADP receptors on platelets, further accelerating platelet aggregation [12–14]. The ADP receptors expressed on the surface of platelets are mainly P2Y1 and P2Y12 [15]. P2Y1 is a G protein-coupled transmembrane receptor and the first receptor to be cloned. When ADP binds to P2Y1 receptor, P2Y1 receptor and Gq protein are coupled to activate phosphatase C, which leads to Ca2+ flowing into the cell from outside the cell. The intracellular Ca2+ concentration increases, integrin *α*IIb*β*3 activates, and platelet deformation and gathering occur [16]. P2Y12, also known as P2T, P2Yac, and SPl999, is a class of G protein-coupled receptors involved in ADP-induced platelet aggregation and plays an important role in ADP-mediated thromboxane A2 production. The P2Y12 receptor can also inhibit the expression of adenylate cyclase (AC) by binding to Gi protein and reduce the concentration of cyclic adenosine monophosphate (cAMP), while activating *β* and *γ* of PI3K eventually activates guanosine triphosphatase, causing or accelerating platelet aggregation [17, 18]. P2Y12 receptor antagonists can attenuate ADP-induced platelet aggregation. Currently, anti-ADP receptor drugs are mainly targeted at the P2Y12 receptor.

In recent years, the role of the PI3K-Akt signaling pathway in thrombosis and platelet aggregation has received increasing attention [19–22]. Phosphatidylinositol 3-kinase (PI3K) is a class of lipid kinases involved in intracellular signaling [23]. PI3K can be mainly divided into three types: type I, type II, and type III. At present, there are many types of research on type I PI3K. Type I PI3K can be further divided into four subtypes, PI3K*α*, PI3K*β*, PI3K*δ*, and PI3K*γ*, which are closely related to the tumor, thrombus, and immune and inflammatory processes. Studies have shown that PI3Kp110*β* plays an important role in platelet accumulation and thrombosis. PI3Kp110*β* selective inhibitors can effectively inhibit the formation of thrombus without affecting the normal hemostasis process [24–26]. We observed the effect of FSAMB on the expression of PI3Kp110*β* protein. The results showed that the expression of PI3Kp110*β* was significantly increased by ADP-stimulated platelet activation, and FSAMB intervention significantly inhibited ADP-induced PI3Kp110*β* protein expression. Akt is a signaling molecule downstream of PI3K and is widely recognized as an activation marker for the PI3K/Akt signaling pathway. Activation of PI3K leads to Akt phosphorylation, which plays an important role in platelet aggregation and thrombosis. Therefore, the PI3K/Akt signaling pathway has become an emerging target for the treatment of platelet-related diseases [27–29]. Our study found that FSAMB can inhibit ADP-induced Akt phosphorylation in a dose-dependent manner, indicating that FSAMB exerts antiplatelet aggregation by inhibiting PI3K-Akt signaling pathway. Further analysis of PI3K activity revealed that FSAMB and PI3K inhibitors could synergistically inhibit Akt phosphorylation. This result further confirms that FSAMB has a clear inhibitory effect on the PI3K-Akt signaling pathway.

The integrin receptor *α*IIb*β*3 participates in two signaling pathways in platelets through binding to various adhesion ligands, namely, the “inside-out” signaling pathway and the “outside-in” signaling pathway [30, 31]. Platelet aggregation reflects the “inside-out” signal, and the above experiments have confirmed that FSAMB can regulate this signal. The expansion of platelets on fibrinogen can reflect FSAMB's regulation of the outside-in signal. Our results show that FSAMB can significantly inhibit the expansion of platelets on fibrinogen. That is, FSAMB can effectively inhibit both inside-out and outside-in *α*IIb*β*3-mediated processes, both of which are regulated by PI3K [24, 25], suggesting that FSAMB may exert antiplatelet effects through PI3K/Akt.

In summary, FSAMB can inhibit ADP-induced platelet aggregation and inhibit platelet expansion. The potential molecular mechanism of the antiplatelet effect of FSAMB may be related to the inhibition of the PI3K/Akt signaling pathway, which will provide experimental evidence for the prevention and treatment of cardiovascular and cerebrovascular diseases.

## Figures and Tables

**Table 1:** FSAMB inhibited ADP-induced platelet aggregation in vitro and vivo. ^*※*^Platelet aggregation rate is abbreviated as PAR which is intervened by ADP 10umol/L. Mean ± SD. n = 8.  ^*∗*^P<0.05 and ^*∗∗*^P<0.01 versus the model control group. ^#^P<0.05 and ^##^P<0.01 versus drug control.

Group	n	PAR^*※*^	PAR^*※*^	LDH	CK-MB	cTnI
		In vitro (%)	In vivo (%)	(U/L)	(U/L)	(ng/mL)
Normal control (NaCl 0.15mol/L)	8	59.99±4.45^*∗∗*^	48.41±4.16^*∗∗*##^	309±26^*∗∗*##^	3.49±0.56^*∗∗*##^	2.93±0.38^*∗∗*##^
Model control (ADP10umol/L)	8	73.14±4.13^##^	61.23±5.38^##^	1923±327^##^	15.83±3.68^##^	13.55±2.67
FSAMB (168mg/kg)	8	58.75±3.92^*∗∗*^	50.05±4.50^*∗∗*##^	1800±318^*∗∗*##^	14.38±2.16^#^	13.25±2.27
FSAMB (336mg/kg)	8	57.21±3.26^*∗∗*#^	33.60±3.24^*∗∗*##^	1684±283^*∗∗*##^	12.1±3.23^*∗∗*^	12.09±2.13
FSAMB (672mg/kg)	8	50.99±2.93^*∗∗*##^	22.90±3.12^*∗∗*^	1317±169^*∗∗*^	11.37±1.61^*∗∗*^	11.06±2.1^*∗*^
Drug Control	8	61.84±5.26^*∗∗*^ (aspirin)	20.81±1.55^*∗∗*^ (clopidogrel)	1155±280^*∗∗*^	11.42±2.11^*∗∗*^	12.03±1.93

**Table 2:** FSAMB inhibited rat platelet spreading in immobilized fibrinogen. Mean ± SD.  ^*∗*^ P<0.05 and ^*∗∗*^P<0.01 versus model control group.

Group	n	Spreading ratio
Model control (normal saline)	8	30.51±4.08
FSAMB (168mg/kg)	8	16.09±2.36^*∗∗*^
FSAMB (336mg/kg)	8	12.32±2.14^*∗∗*^
FSAMB (672mg/kg)	8	10.13±1.40^*∗∗*^

**Table 3:** FSAMB inhibited PI3K expression and Akt phosphorylation in ADP- (10*μ*mol /L) stimulated platelets. R: resting platelets. Mean ± SD. n =8.  ^*∗*^P<0.05 and ^*∗∗*^P<0.01 versus model control group.

Group	n	PI3K protein expression ratio (%)	Akt phosphorylation ratio (%)
R	8	29.25±4.85	3.93±0.72
Model control (ADP 10umol/L)	8	100±0.00	100±0.00
FSAMB (168mg/kg)	8	46.68±5.27^*∗∗*^	36.26±4.60^*∗∗*^
FSAMB (336mg/kg)	8	39.25±3.60^*∗∗*^	25.22±3.55^*∗∗*^
FSAMB (672mg/kg)	8	30.04±5.50^*∗∗*^	15.34±2.34^*∗∗*^

**Table 4:** Effect of PI3K inhibitor LY294002 on Akt phosphorylation. R: resting platelets. Mean ± SD. n= 8. ^*∗∗*^P<0.01 versus model control group; ^##^P<0.01 versus FSAMB (336mg/kg) group.

Group	n	Akt phosphorylation ratio (%)
R	8	3.93±0.72
Model control (ADP 10umol/L)	8	100±0.00
FSAMB (336mg/kg)	8	36.26±4.60^*∗∗*^
LY294002 (5umol/L)	8	25.22±3.55^*∗∗*^
LY294002 (5umol/L) + FSAMB (336mg/kg)	8	15.34±2.34^*∗∗*##^

## Data Availability

The datasets generated and/or analyzed during the current study are available from the corresponding author upon reasonable request.
